# Supplementary material for: Development-Related miRNA Expression and Target Regulation during Staggered In Vitro Plant Regeneration of Tuxpeño VS-535 Maize Cultivar
Source: Int J Mol Sci. 2019 Apr 27;20(9):2079. doi: 10.3390/ijms20092079 (PMC6539278; doi:10.3390/ijms20092079)
Supplement: Supplementary file 1 [file ijms-20-02079-s001.pdf]

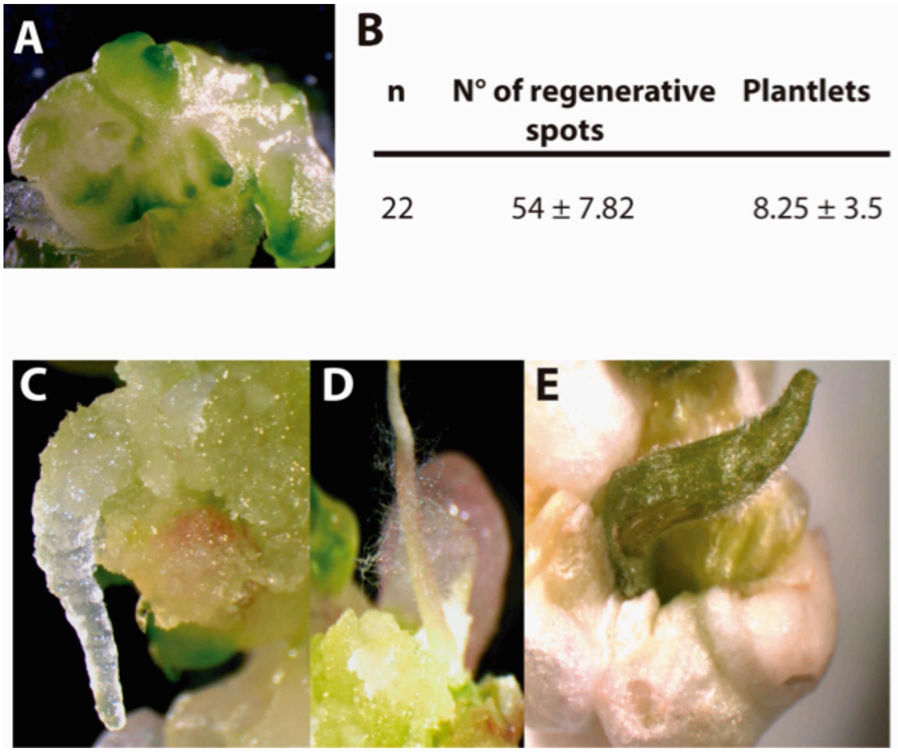

n	N° of regenerative spots	Plantlets
22	54 ± 7.82	8.25 ± 3.5

Figure S1. **A**. Callus with several regenerative spots on its surface. **B**. Number of regenerative spots per 22 replicates (22 culture jars, each one with ~1 g of calli) and number of fully regenerated plantlets. **C**. Root-like formation observed on Y-NEC in N6P with 50% hormones. **D**. Fully developed root on Y-NEC in N6P with 0% hormones. **E**. W-NEC in MS showing regeneration of aberrant leaf structures, which do not achieve complete plantlet regeneration.

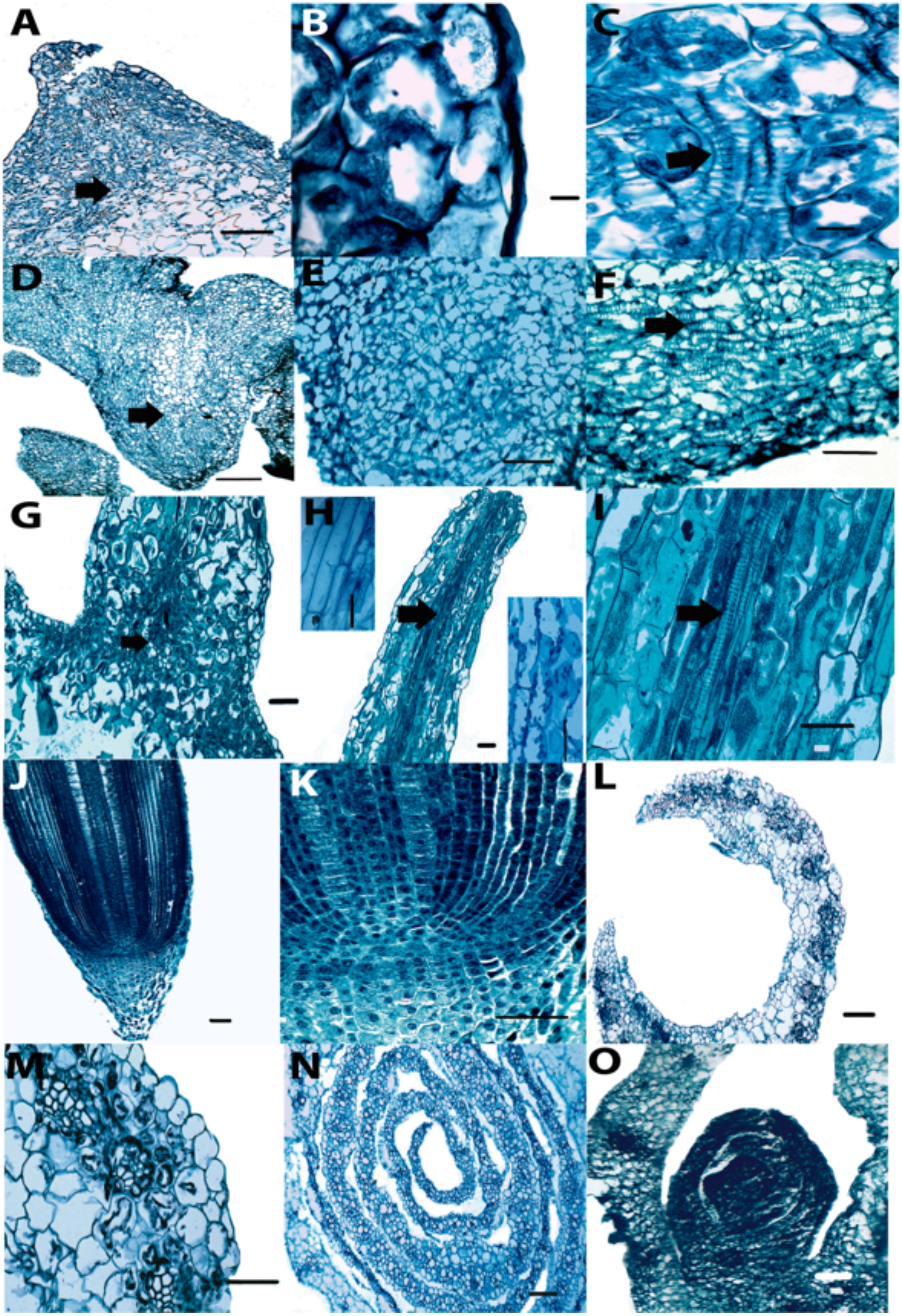
A
B
C
D
E
F
G
H
I
J
K
L
M
N
O

Figure S2. **A.** Longitudinal section of the 1st stage of development (X400 magnification). The arrowhead indicates tracheid structures amplified in C. **B.** Meristematic cells present in the regenerative spot (X1000 magnification). **C.** Tracheid structures observed at the base of regenerative spots (X1000 magnification). **D.** Longitudinal section of the 2nd stage of development (X100 magnification). The arrowhead indicates tracheid structures amplified in F. **E.** Proliferative portion of the 2nd stage showing meristematic cells (X400 magnification). **F.** Tracheid structures from D (X400 magnification). **G.** Longitudinal section of a basal leaf portion, 3rd stage of development (X100 magnification). Arrow indicated aligned tracheid. **H.** Longitudinal section of a tip leaf portion, 3rd stage of development (X100 magnification). The insets show chlorenchyma (right) and amyloplasts (left) observed in the section. **I.** Aligned tracheid structures highlighted by an arrow are present at the 3rd stage (X400 magnification). **J and K.** Longitudinal section of a plantlet root at X100 and X400 magnifications, respectively. The root apical meristem (RAM) is observed. **L and M.** Cross section of a plantlet leaf at X100 and X400 magnifications, respectively. Vascular bundles are appreciated. **N.** Cross section of a plantlet stem showing no evidence of central vascular bundles. **O.** SAM and leaf primordium observed in a transversal section of a plantlet. Bar: 100 µm, except in B: 20 µm.

Summary against regulator

Export all data as SIF file ⇩

	leaf	root	SAM	seed	
**GRMZM2G126018**	765	28	34	71	SBP23
**GRMZM2G159399**	130	1350	603	333	ARF17
**GRMZM2G393433**	0	90	782	0	CUC2
**GRMZM2G109987**	2290	1505	1128	1963	RLD1
**GRMZM2G064954**	0	0	0	0	F-Box
**GRMZM2G028622**	0	0	0	0	WUS2
**AC207656.3_FGT002**	0	0	0	0	ARF19

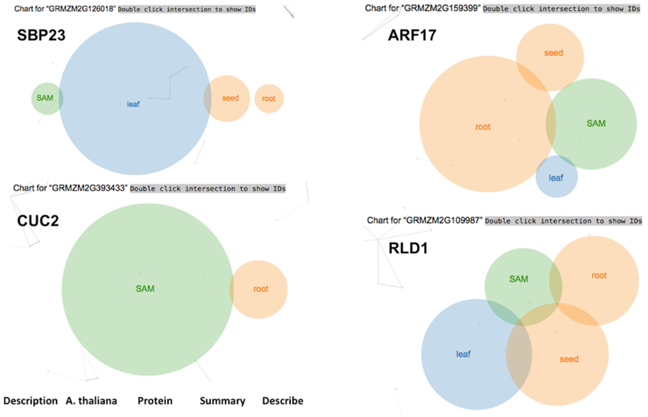

Regulators	Targets	Symbol	Chr.	5'position	3'position	Description	A. thaliana	Protein	Summary	Describe
**SAM(24)**										
GRMZM2G126018	GRMZM2G065171		2	43429634	43434223	Uncharacteri	AT5G10050.1	NAD(P)-binding Rossmann		NAD(P)-binding Rossmann-fold superfamily protein; FUNCTIONS IN: oxidoreductase activity, binding, catal
GRMZM2G126018	GRMZM2G129238		2	219135240	219132473		AT3G08510.2	phospholipa:	Phosphoinos	phospholipase C 2 (PLC2); FUNCTIONS IN: phospholipase C activity; INVOLVED IN: signal transduction, intra
GRMZM2G126018	GRMZM2G171880		2	201062180	201069661	Uncharacteri	AT4G38050.1	Xanthine/uracil permease		Xanthine/uracil permease family protein; FUNCTIONS IN: transmembrane transporter activity; INVOLVED I
GRMZM2G126018	GRMZM2G085420		3	159653178	159647652		AT1G48830.2	Ribosomal protein S7e fan		Ribosomal protein S7e family protein; FUNCTIONS IN: structural constituent of ribosome; INVOLVED IN: tra
GRMZM2G126018	GRMZM2G080156		2	16043158	16034837	Uncharacteri	AT2G16530.2	3-oxo-5-alpha-steroid 4-de		3-oxo-5-alpha-steroid 4-dehydrogenase family protein; FUNCTIONS IN: oxidoreductase activity, acting on t
GRMZM2G126018	GRMZM2G378949		4	96452068	96455783	Uncharacteri	AT2G41140.1	CDPK-relatec	Encodes CDP	CDPK-related kinase 1 (CRK1); FUNCTIONS IN: calcium-dependent protein serine/threonine phosphatase a
GRMZM2G159399	GRMZM2G054225	nrpd2/e2	2	6347954	6336518	DNA-directe	AT3G23780.2	nuclear RNA	This gene en	nuclear RNA polymerase D2A (NRPD2A); CONTAINS InterPro DOMAIN/s: DNA-directed RNA polymerase, su
GRMZM2G159399	GRMZM2G077356	iaa21	5	7780495	7783441	Uncharacteri	AT3G04730.1	indoleacetic	early auxin-ir	indoleacetic acid-induced protein 16 (IAA16); FUNCTIONS IN: sequence-specific DNA binding transcription
GRMZM2G159399	GRMZM2G161368		1	213136622	213138903	Selenium-bir	AT3G47530.1	Pentatricopeptide repeat (		Pentatricopeptide repeat (PPR) superfamily protein; CONTAINS InterPro DOMAIN/s: Pentatricopeptide rep
GRMZM2G159399	AC215198.3_FG002	ppr2263	9	12723540	12725903	Pentatricope	AT4G30700.1	Pentatricope	Encodes a pe	Pentatricopeptide repeat (PPR) superfamily protein; CONTAINS InterPro DOMAIN/s: Pentatricopeptide rep
GRMZM2G159399	GRMZM2G102639		5	31202697	31198276	Uncharacteri	AT2G18510.1	RNA-binding (RRM/RBD/R		embryo defective 2444 (emb2444); FUNCTIONS IN: RNA binding, nucleotide binding, nucleic acid binding;
GRMZM2G159399	GRMZM2G357399		3	186637449	186634201	ADP-ribosyla	AT3G62290.3	ADP-ribosyla	A member of	ADP-ribosylation factor A1E (ARFA1E); CONTAINS InterPro DOMAIN/s: ADP-ribosylation factor (InterPro:IP
GRMZM2G393433	GRMZM2G139700	nactf84	3	137606842	137608651	Putative uncl	AT5G53950.1	NAC (No Api	Transcriptior	CUP-SHAPED COTYLEDON 2 (CUC2); CONTAINS InterPro DOMAIN/s: No apical meristem (NAM) protein (In
GRMZM2G393433	GRMZM2G085113	te1	3	165220769	165216844	Protein term	AT3G26120.1	terminal EAR	Similar to ter	terminal EAR1-like 1 (TEL1); FUNCTIONS IN: RNA binding, nucleotide binding, nucleic acid binding; EXPRESS
GRMZM2G393433	GRMZM2G049151		7	152844283	152843404	Uncharacteri	AT4G04630.1	Protein of unknown functi		Protein of unknown function, DUF584; CONTAINS InterPro DOMAIN/s: Protein of unknown function DUF5
GRMZM2G393433	GRMZM2G167986	cyp8	1	243707108	243709449	Cytochrome	AT5G09970.1	cytochrome	member of C	cytochrome P450, family 78, subfamily A, polypeptide 7 (CYP78A7); FUNCTIONS IN: electron carrier activit
GRMZM2G393433	GRMZM2G142721		7	4686349	4685362	G1 protein [	AT1G07090.1	Protein of unknown functi		LIGHT SENSITIVE HYPOCOTYLS 6 (LSH6); CONTAINS InterPro DOMAIN/s: Protein of unknown function DUF
GRMZM2G393433	GRMZM2G180190	zfl2	2	12652213	12649206	Floricaula/le	AT5G61850.1	floral meriste	Encodes tran	LEAFY (LFY); CONTAINS InterPro DOMAIN/s: Floricaula/leafy protein (InterPro:IPR002910); Has 1807 Blast
GRMZM2G109987	GRMZM2G042250	rld2	1	2791599	2798512	Rolled leaf 2	AT5G60690.1	Homeobox-le	REVOLUTA re	REVOLUTA (REV); CONTAINS InterPro DOMAIN/s: Homeobox (InterPro:IPR001356), Homeodomain-like (In
GRMZM2G109987	GRMZM2G117222		1	70254818	70262426	FUNCTIONS I	AT1G19140.2			FUNCTIONS IN: molecular_function unknown; INVOLVED IN: ubiquinone biosynthetic process; LOCATED IN
GRMZM2G109987	GRMZM2G130375		3	220735848	220743609	Uncharacteri	AT3G13750.1	beta galactos	beta-galacto:	beta galactosidase 1 (BGAL1); FUNCTIONS IN: beta-galactosidase activity; INVOLVED IN: carbohydrate met
GRMZM2G109987	GRMZM2G441583	ago1a	6	43253105	43261555	Putative argc	AT1G48410.1	Stabilizer of i	Encodes an F	ARGONAUTE 1 (AGO1); FUNCTIONS IN: protein binding, endoribonuclease activity, siRNA binding, miRNA b
GRMZM2G109987	GRMZM5G844195		5	3841759	3850283	Putative uncl	AT3G51310.1	VPS35 homo	Homolog of y	VPS35 homolog C (VPS35C); FUNCTIONS IN: molecular_function unknown; INVOLVED IN: intracellular prot
GRMZM2G109987	GRMZM5G886363	brk3	10	2017183	2011814	Uncharacteri	AT2G35110.2	transcription	Component c	GNARLED (GRL); CONTAINS InterPro DOMAIN/s: Nck-associated protein 1 (InterPro:IPR019137); Has 3533

Figure S3. Gene regulatory network for miRNA-targeted transcription factors (TF) analyzed during *in vitro* maize plant regeneration. miRNA targets were tested using a publicly available database for maize tissue-specific gene regulatory networks (Huang et al., 2018; https://www.bio.fsu.edu/mcginnislab/mgrn/). Data for SBP23 (GRMZM2G126018), ARF17 (GRMZM2G159399), CUC2 (GRMZM2G393433) and RLD1 (GRMZM2G109987) targets were successfully retrieved from the database in four tissues: seed, SAM, leaf and root. CUC2 targets were found only for SAM and root tissues. A summary table for all TF targets and screenshots of interactive tissue Venn diagrams for these targets are shown in the upper part. A table showing the top 6 targets in SAM for each TF is displayed at the bottom.

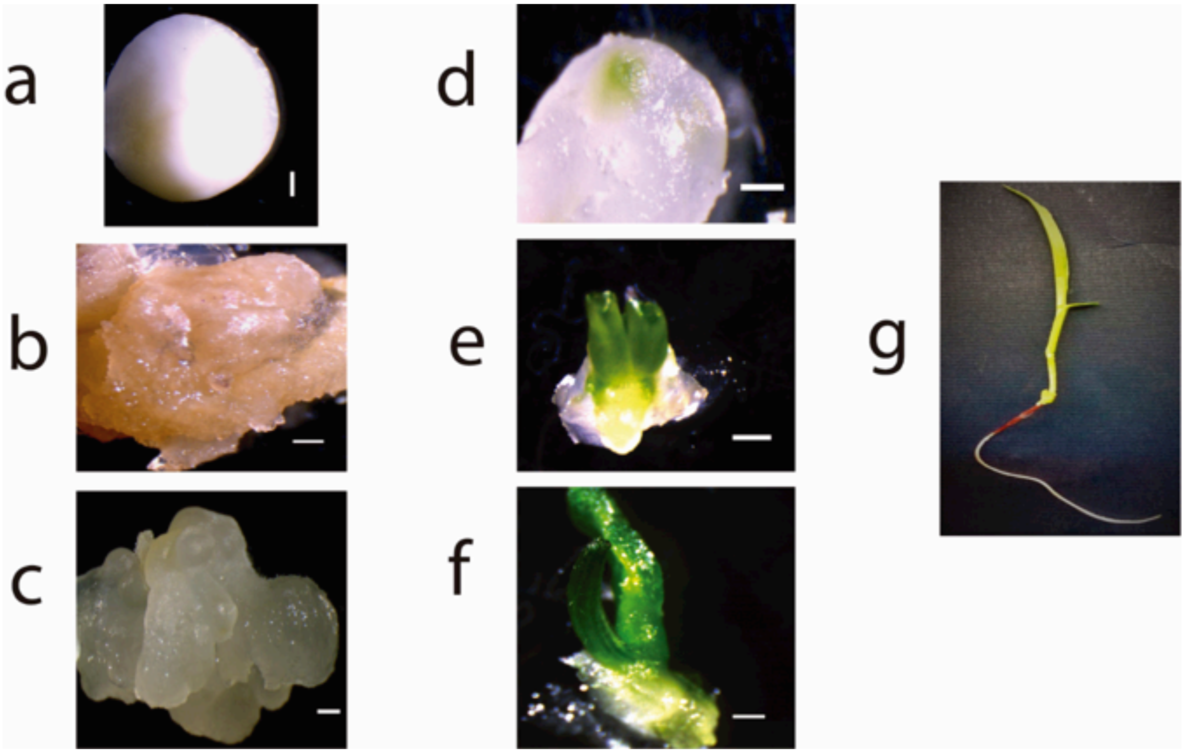

Figure S4. Tissues used for molecular analysis. **(a)** Immature Embryo, **(b)** Y-NEC, **(c)** EC, **(d)** 1st stage of development, **(e)** 2nd stage of development, **(f)** 3rd stage of development, **(g)** Plantlet.

Table S1. Medium composition.

Composition of N6P medium		Composition of MS medium	
**Component**	**g/L**	Component	**g/L**
KNO_3	2.83	KNO_3	1.9
$(NH_4)_2SO_4$	0.463	NH_4NO_3	1.65
KH_2PO_4	0.4	$CaCl_2.2H_2O$	0.44
$MgSO_4.7H_2O$	0.185	$MgSO_4.7H_2O$	0.37
$CaCl_2.2H_2O$	0.166	$Na_2MoO_4{\cdot}2H_2O$	0.25
$Na_2EDTA.H_2O$	0.0375	KH_2PO_4	0.17
$FeSO_4.7H_2O$	0.0278	$Na_2EDTA.2H_2O$	0.0373
$MnSO_4.H_2O$	0.00332	$FeSO_4.7H_2O$	0.0278
H_3BO_3	0.0016	$MnSO_4.4H_2O$	0.0223
$ZnSO_4.7H_2O$	0.0015	H_3BO_3	0.0062
KI	0.00083	$ZnSO_4.4H_2O$	0.0086
Adenine	0.1	KI	0.00083
Proline	2.5	$CoCl_2.2H_2O$	0.000025
Casein	0.200	$CuSO_4{\cdot}5H_2O$	0.000025
Sucrose	30	Inositol	0.1
2,4 Dichlorophenoxyacetic *acid*	0.002	Nicotinic acid	0.001
6-BA (6-benzyladenine)	0.0003	Thiamine	0.002
Inositol	0.1	Pyridoxine	0.001
Nicotinic acid	0.0005	Glycine	0.002
Thiamine	0.0001	Sucrose	30
Pyridoxine	0.0005	Agargel	7
Agargel	7		

Table S2. Oligonucleotides used in this study. miRNA sequences were retrieved from miRBase (http://www.mirbase.org) and Target cDNA sequences from Maize Genetics and Genomic Database (https://www.maizegdb.org/).

**ID**		**SEQUENCE (5' → 3')**
**U6 snRNA**	Primer stem loop	GTGCAGGGTCCGAGGTTTTGGACCATTTCTCGAT
	Forward primer	GGAACGATACAGAGAAGATTAGCA
**zma-miR156a-5p***	Primer stem loop	GTCGTATCCAGTGCAGGGTCCGAGGTATTCGCACTGGATACG ACGTGCTC
	Forward primer	TGCTCGTGACAGAAGAGAGT
**zma-miR160a-5p**	Primer stem loop	GTCGTATCCAGTGCAGGGTCCGAGGTATTCGCACTGGATACGA CTGGCAT
	Forward primer	TTTGCCTGGCTCCCTGT
**zma-miR164a-5p**	Primer stem loop	GTCGTATCCAGTGCAGGGTCCGAGGTATTCGCACTGGATACG ACTGCACG
	Forward primer	CTACTGGAGAAGCAGGGCA
**zma-miR166b-3p**	Primer stem loop	GTCGTATCCAGTGCAGGGTCCGAGGTATTCGCACTGGATACGA CGGGAAT
	Forward primer	CGTCGCTCGGACCAGGCTTCA
**zma-miR394a-5p**	Primer stem loop	GTCGTATCCAGTGCAGGGTCCGAGGTATTCGCACTGGATACGA CGGAGGT
	Forward primer	TTCGTTTGGCATTCTGTCCA
**Universal reverse primer**		GTGCAGGGTCCGAGGTA
**SPB23** (GRMZM2G126018_T01)**		Forward ACACCAACGCGATGAATTGG<br>Reverse ACCCTGAAAAACCAGAACGG
**ARF17** (GRMZM2G159399_T01)		Forward TTTCTCGGACATCGCTCCTG<br>Reverse CCTTGGATATACGGGGCGTC
**ARF19** (AC207656.3_FGT002)		Forward TCCCACTGTACCCGGAGCTT<br>Reverse GCATGCCTGGCTCCCTGTAT
**CUC2** (GRMZM2G393433_T01)		Forward TTCGCTGCACTACATGGTTG<br>Reverse AACGACGACCCAGTCACTTAC
**RDL1** (GRMZM2G109987_T01)		Forward GCGATTGCAGAGGAGACCTT<br>Reverse TGGCCACGATACCAACTGAA
**F-BOX** (GRMZM2G064954 _T01)		Forward GATGACATGCCTGGGCAACA<br>Reverse GCTTTTTGCGGCTGTATGGTA
**WUS2** (GRMZM2G02862)		Forward TTTACAGCAACAGCACCCAG<br>Reverse CAGGGTAAGGGGAGCACCAT
**18S** (XM_020546348.1)		Forward TCCTATTGTTGGCCTTCGG<br>Reverse TCCTTGGCAAATGCTTTCGC
